# Correction: Acute kidney injury in patients with Visceral Leishmaniasis in Northwest Ethiopia

**DOI:** 10.1371/journal.pone.0306663

**Published:** 2024-07-02

**Authors:** Workagegnehu Hailu, Rezika Mohamed, Helina Fikre, Saba Atnafu, Azeb Tadesse, Ermias Diro, Johan van Griensven

The seventh author’s name is spelled incorrectly. The correct name is: Johan van Griensven.
